# Better Health Information

**Published:** 1920-02-21

**Authors:** 


### Better Health Information.

The Ministry of Health have taken a useful step in the direction of securing wider and better health information. They have called upon local authorities to instruct their medical officers of health to compile their annual reports for 1919, and they urge the discussion from each district of natural and social conditions; sanitary circumstances and administration; food supplies; prevalence of, and control over, infectious diseases; maternity and child welfare; and housing and other services.

It is pointed out that such a scrutiny has become doubly requisite in order to bring to light any local consequences of war conditions that may need special attention, and to form the basis for a comprehensive scheme of health developments generally, which, it is hoped, may shortly follow upon the recent unification in the Ministry of Health of the various central functions in respect of all matters affecting public health.

The Ministry suggests a regular system of exchange of such annual reports between neighbouring authorities and also of areas in other parts of the kingdom whose conditions are more or less comparable.

In the past the tendency has been for authorities to work too much in isolation, and the Ministry points out the loss of opportunities for progress which results from failures to "pool experiences for the common good."

The following paragraph deserves special attention as advising a course of action which is at the root of all successful public-health work :—

"It is further suggested that your council should arrange for the report to be distributed locally, as soon as it is available, as widely as possible, and should take steps through the local press and otherwise to bring its contents effectively to the knowledge of the people. One of the main purposes of the compilation of the report is that, by giving the widest possible publicity, it shall engender a popular interest in the subject, and an enlightened public opinion which shall support the local authority in realising its high responsibilities for the health of its area, and in remedying, at the earliest opportunity, the various defects which the survey may bring to light, whether arising from war conditions, or from other causes. Such an increase of public knowledge and interest in these matters may also become an effective means of educating the citizens in the more important conditions of public health, of warning them against particular dangers and of securing that highly important co-operation and confidence between them and the health authority and its staff which is essential to successful health administration."

